# Impact of stillbirths on international comparisons of preterm birth rates: a secondary analysis of the WHO multi‐country survey of Maternal and Newborn Health

**DOI:** 10.1111/1471-0528.14548

**Published:** 2017-02-20

**Authors:** N Morisaki, T Ganchimeg, JP Vogel, J Zeitlin, JG Cecatti, JP Souza, C Pileggi Castro, MR Torloni, E Ota, R Mori, SM Dolan, S Tough, S Mittal, V Bataglia, B Yadamsuren, MS Kramer, on behalf of the PREBIC Epidemiology Working Group and the WHO‐MCS Research Network

**Affiliations:** ^1^ Department of Social Medicine National Center for Child Health and Research Tokyo Japan; ^2^ Department of Global Health Nursing Faculty of Medicine University of Tsukuba Tsukuba Ibaraki Japan; ^3^ UNDP/UNFPA/UNICEF/WHO/World Bank Special Programme of Research, Development and Research Training in Human Reproduction (HRP) Department of Reproductive Health and Research World Health Organization Geneva Switzerland; ^4^ Perinatal and Pediatric Epidemiology Research Team Center for Epidemiology and Statistics Sorbonne Paris Cité, DHU Risks in Pregnancy Paris Descartes University Paris France; ^5^ Department of Obstetrics and Gynecology School of Medical Sciences University of Campinas Campinas Brazil; ^6^ Department of Social Medicine Ribeirao Preto Medical School University of Sao Paulo Ribeirao Preto Sao Paulo Brazil; ^7^ Department of Pediatrics Ribeirao Preto Medical School University of Sao Paulo Ribeirao Preto Sao Paulo Brazil; ^8^ Evidence Based Healthcare Post‐graduate Program Sao Paulo Federal University Sao Paulo Brazil; ^9^ Global Health Nursing Graduate School of Nursing Science St. Luke's International University Tokyo Japan; ^10^ Department of Health Policy National Center for Child Health and Development Tokyo Japan; ^11^ Department of Obstetrics & Gynecology and Women's Health Albert Einstein College of Medicine/Montefiore Medical Center New York NY USA; ^12^ Departments of Paediatrics and Community Health Sciences Cumming School of Medicine University of Calgary Calgary AB Canada; ^13^ Department of Obstetrics & Gynecology Fortis Memorial Research Institute Gurgaon India; ^14^ Department of Gynaecology, Obstetrics and Perinatology Central Hospital Social Security Institute Asuncion Paraguay; ^15^ National Center for Communicable Diseases Ulaanbaatar Mongolia; ^16^ Departments of Pediatrics and of Epidemiology, Biostatistics and Occupational Health McGill University Faculty of Medicine Montreal QC Canada

**Keywords:** Global health, low income country, perinatal health, preterm birth, stillbirth

## Abstract

**Objective:**

To evaluate the extent to which stillbirths affect international comparisons of preterm birth rates in low‐ and middle‐income countries.

**Design:**

Secondary analysis of a multi‐country cross‐sectional study.

**Setting:**

29 countries participating in the World Health Organization Multicountry Survey on Maternal and Newborn Health.

**Population:**

258 215 singleton deliveries in 286 hospitals.

**Methods:**

We describe how inclusion or exclusion of stillbirth affect rates of preterm births in 29 countries.

**Main outcome measures:**

Preterm delivery.

**Results:**

In all countries, preterm birth rates were substantially lower when based on live births only, than when based on total births. However, the increase in preterm birth rates with inclusion of stillbirths was substantially higher in low Human Development Index (HDI) countries [median 18.2%, interquartile range (17.2–34.6%)] compared with medium (4.3%, 3.0–6.7%), and high‐HDI countries (4.8%, 4.4–5.5%).

**Conclusion:**

Inclusion of stillbirths leads to higher estimates of preterm birth rate in all countries, with a disproportionately large effect in low‐HDI countries. Preterm birth rates based on live births alone do not accurately reflect international disparities in perinatal health; thus improved registration and reporting of stillbirths are necessary.

**Tweetable abstract:**

Inclusion of stillbirths increases preterm birth rates estimates, especially in low‐HDI countries.

## Tweetable abstract

Inclusion of stillbirths increases preterm birth rates estimates, especially in low‐HDI countries.

## Introduction

Approximately 15 million infants are born preterm (<37 completed weeks of gestation) each year.^1^ Preterm birth contributes to over one‐third of the world's estimated 3 million annual neonatal deaths.^2^, ^3^, ^4^ Even after surviving the neonatal period, infants born preterm are at increased risk for delayed childhood development and low economic productivity.^5^ Acknowledgement of the large public health impact of preterm birth has led to many preventive initiatives, including comprehensive antenatal care, childbirth services and emergency obstetric care.^6^, ^7^, ^8^ The preterm birth rate serves as an important population perinatal health indicator for evaluating the success of these initiatives within countries, and for comparisons among countries and regions.

At the same time, more than 2.6 million stillbirths occur per year.^9^ Compared with other indicators of perinatal health, the stillbirth rate has long been neglected.^10^ Only recently has its importance been acknowledged; the World Health Organization now includes it in ‘100 Core Health Indicators’,^11^ and the World Health Assembly has targeted the reduction of stillbirths to below 12 per 1000 total births in every country by 2030.^1^

Although WHO defines preterm birth as any birth before 37 weeks of pregnancy,^3^, ^12^, ^13^ stillbirths are usually excluded when calculating and reporting preterm birth rates, and hence in international comparisons.^3^, ^12^ However, in populations where the burden of stillbirths is large, such exclusions may limit the utility of the preterm birth rate as a robust perinatal indicator. The majority of stillbirths occur before term,^14^, ^15^ and major causes of stillbirth and preterm birth (such as maternal malnutrition and infection) overlap.^10^, ^11^ Preterm delivery rates might therefore be larger in populations with high stillbirth rates if stillbirths were included when calculating preterm birth rates. Additionally, improved provision of obstetric interventions to prevent stillbirth often involve an increase in provider‐initiated early delivery.^16^ Further complications include the non‐registration of stillbirths and the misclassification of early neonatal deaths as stillbirths.^17^, ^18^, ^19^, ^20^ Such factors could result in an artefactual reduction in the reported preterm birth rate, which would not necessarily reflect better perinatal care. However, this issue has been largely ignored, particularly in middle‐ and low‐income countries.

In this study, we use data from the WHO Multicountry Survey on Maternal and Newborn Health to assess the extent to which inclusion or exclusion of stillbirths contributes to differences in reported preterm birth rates among a large group of countries following a common study protocol.

## Methods

### Data source

We conducted a secondary analysis of data from the World Health Organization Multicountry Study on Maternal and Newborn Health (WHOMCS), whose methods have been detailed elsewhere.^21^, ^22^ WHOMCS is a cross‐sectional study whose primary aim was to collect information on maternal deaths and ‘near‐miss’ cases (women with severe complications during pregnancy or delivery who nearly died but survived) among all deliveries from a sample of health facilities in Africa, Asia, Latin America and the Middle East. A stratified, multistage cluster sampling strategy was used to select participating health facilities. From each country, the capital city and two randomly selected provinces (with sampling probability proportional to population) were sampled in each of the 29 participating countries. From a list of all facilities in each sampled jurisdiction with at least 1000 deliveries per year and the capacity to perform caesarean delivery, up to seven facilities were selected for participation (with sampling probability proportional to number of deliveries). A total of 359 health facilities were selected. Trained health staff at those facilities used a standardised form to collect data directly from the medical records of all women who were admitted for delivery or presented with severe maternal outcomes during the study period, irrespective of gestational age or site of delivery. Facilities collected data for 2–4 months between May 2010 and December 2011. All eligible women's data on demographic and reproductive characteristics, pregnancy outcomes, maternal and newborn morbidity and mortality and their management were collected before hospital discharge, death, or the eighth postpartum day, whichever came first.

### Study population

**Figure 1:**
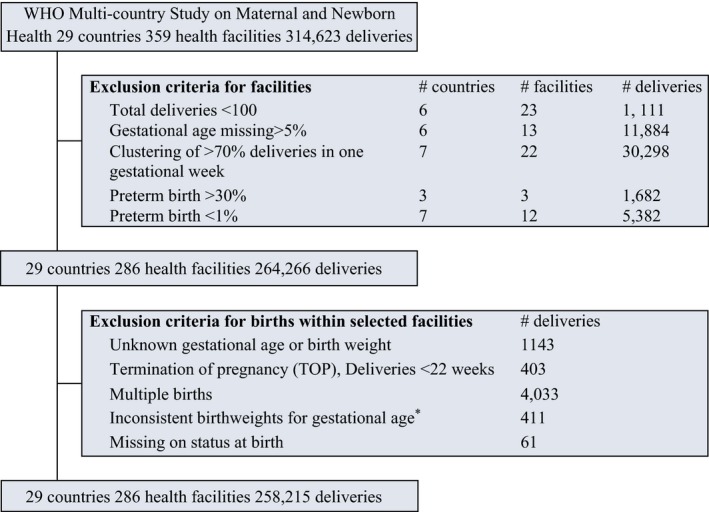
Study population flow chart. *Used Alexander et al. US reference for fetal growth.

We restricted our analysis to women who had singleton livebirth or stillbirth deliveries of at least 22 weeks gestational age. We first identified health facilities with reliable assessment of gestational age and then identified individual births with plausible gestational age assessment when assessed against birthweight (see Figure 1).^23^ To achieve this, we excluded facilities at which gestational age data were missing for >5% of all deliveries and those with an unreliable distribution of gestational age: facilities at which more than 70% of all deliveries occurred at a specific week, or at which more than 30% or less than 1% of all deliveries were preterm. We also excluded facilities with <100 total deliveries. A total of 286 health facilities remained of the total 359.

At the individual level, from the 264 266 records at the 286 health facilities, we excluded termination of pregnancy (TOP), births of less than 22 completed weeks’ gestation, multiple births, births with missing or inconsistent birthweight for gestational age (using the exclusion criteria proposed by Alexander et al.^23^), and deliveries with missing data for vital status at birth. After these exclusions, 258 215 deliveries from 286 facilities in 29 countries remained and were included in our analysis. Details of excluded facilities and births are shown in Table S1.

### Variables and measurements

In the WHOMCS, gestational age was based on the best obstetric assessment according to local practices. The method used to assess individual gestational age was not recorded and thus may or may not have included the use of ultrasound. Deliveries were coded as livebirth, or stillbirth (macerated or fresh). Live birth was defined according to WHO criteria: complete expulsion or extraction from its mother of a product of conception, irrespective of the duration of the pregnancy, with any evidence of life.

In this secondary analysis, we grouped gestational ages following the WHO preterm birth categorisation:^3^ extremely preterm (22–27 weeks), very preterm (28–31 weeks), moderate preterm (32–33 weeks) or late preterm (34–36 weeks). To investigate variations in live birth registration at very early gestations, we additionally used the sub‐categories of 22–23 weeks and 24–27 weeks. For international comparison, WHO recommends reporting of stillbirths of at least 28 weeks’ gestation. We therefore also estimated overall preterm birth rates in each country, and by human development index (HDI, see below) category, for both 22–36 weeks and 28–36 weeks.

As an indicator of country development, we classified countries according to the United Nations Development Programme HDI ranking in 2012.^24^ The participating countries in the WHOMCS were categorised into three groups: very high or high (HDI ranking 1–100: Japan, Qatar, Argentina, Mexico, Lebanon, Peru, Brazil, Ecuador and Sri Lanka); medium (HDI ranking 101–150: Jordan, China, Thailand, Mongolia, Occupied Territory of Palestine, Paraguay, Philippines, Vietnam, Nicaragua, India and Cambodia); or low (HDI ranking >150: Kenya, Pakistan, Angola, Nigeria, Nepal, Uganda, Afghanistan, Democratic Republic of Congo and Nigeria) (countries are listed in order of HDI).

### Statistical analysis

We first compared the proportion of live births among deliveries in each country, and among the three HDI groups, using the Kruskal–Wallis test for the following gestational age strata: 22–23 weeks, 24–27 weeks, 28–31 weeks, 32–33 weeks and 34–36 weeks. Next, to assess the impact of including or excluding stillbirths on preterm rates, we calculated rates of preterm delivery (22–36 weeks), extreme or very preterm (22–31 weeks) delivery, and moderate and late preterm (32–36 weeks) delivery in each country using two methods:

- Restriction to live births in each gestational age stratum (denominator: all live births ≥22 weeks’ gestation)
- Combined live births and stillbirths in each gestational age stratum (denominator: total of live births plus stillbirths ≥22 weeks’ gestation).

Finally, the change in preterm delivery rates when stillbirths are included was calculated for each country, as well as by HDI category.

As gestational age estimation may be imprecise compared with birthweight in settings where ultrasound is not routinely available, we further conducted a sensitivity analysis based on birthweight categories (<1000, 1000–1499, 1500–1999, 2000–2499, ≥2500 g) and compared the low birthweight rate instead of the preterm birth rate. Our main analysis was limited to singletons, as previous studies have shown that rates of multiple pregnancy (which have much higher preterm birth rates^25^) differ widely among countries^26^, ^27^ and could affect the association of interest^27^; however, we also conducted a sensitivity analysis that included multiple births.

All analyses were conducted using STATA 13.1 MP (Stata Corp, College Station, TX, USA).

## Results

**Figure 2:**
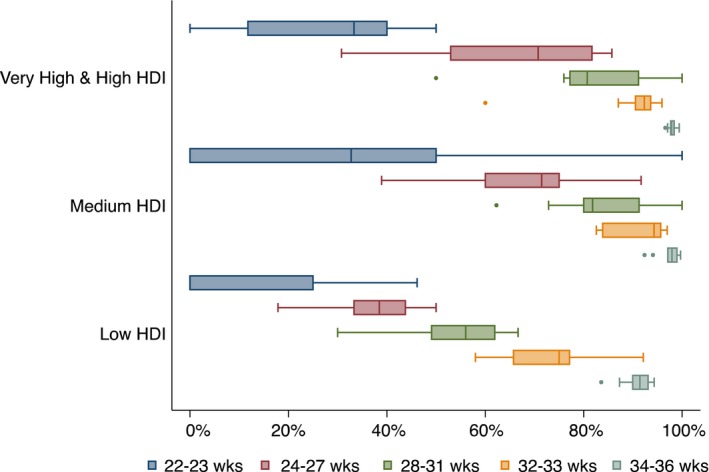
Proportions of live births among all deliveries [live births/(live births + stillbirths)] by gestational age category. Comparison between countries of high‐, medium‐ and low‐Human Developmental Index participating in the WHO Multicountry Survey. Centre lines show the medians; box limits indicate the 25th and 75th percentiles; whiskers extend 1.5 times the interquartile range from 25th and 75th percentiles; circles show outliers.

A total of 236 862 live births were recorded in the 29 countries, 17 004 of which were preterm. At the same time, there were 4349 stillbirths, of which 2144 were preterm. Box plots of proportions of live births among all deliveries (live births + stillbirths) in each country are shown in Figure 2 by HDI level for each gestational age stratum of 22–23, 24–27, 28–31 and 32–36 weeks (country‐specific proportions are shown in Table S2). The proportion of live births increased with higher gestational age in all HDI groups, with median percentages ranging from 0–36.7% at 22–23 weeks to 91.5–97.9% at 34–36 weeks. Proportions of live births for deliveries at 24–27 weeks, 28–31 weeks, 32–33 weeks and 34–36 weeks in medium‐HDI countries were not significantly different from those in high‐HDI countries. In low‐HDI countries, however, proportions of live births were significantly lower than in medium and high‐HDI countries for all gestational age categories except 22–23 weeks (Table S3). A similar association was seen when birthweight categories (<1000, 1000–1499, 1500–1999, 2000–2499 g) were used instead of gestational age categories (Table S4), as well as when multiple births were included (Table S5).

Preterm birth rates calculated including and excluding stillbirths are shown in Table 1. In all countries, preterm birth rates were higher when stillbirths were included. However, when grouped by HDI, the increase in preterm birth rates when including stillbirths was significantly larger in low‐HDI countries [median 18.2%, interquartile range (17.2–34.6%)] compared with medium‐HDI (4.3%, 3.0–6.7%) and very high/high‐HDI countries (4.8%, 4.4–5.5%) (Table 2).The increase was larger in lower gestational age categories, but the disparity between low‐HDI countries and very high/high‐ and medium‐HDI countries was apparent in all gestational age categories. This disparity could also be observed when birthweight categories were used instead of gestational age categories, as well as when multiple births were included in the analysis (Tables S6 and S7).

**Table 1:** Differences in preterm birth rates calculated including and excluding stillbirths among 29 countries participating in the WHO Multicountry Survey

	Countries	Total Deliveries	Preterm deliveries		Term deliveries		Term live birth rate	Preterm birth rate		Increase in preterm birth rate by including stillbirths^a^	Increase in preterm birth rate by including stillbirths (%)^b^
			Live births	Stillbirths	Live births	Stillbirths		Excluding stillbirths	Including stillbirths		
Very high‐HDI	Japan	3496	187	6	3300	3	94.4	5.4	5.5	0.16	+2.9
	Qatar	3879	150	9	3711	9	95.7	3.9	4.1	0.21	+5.5
	Argentina	9599	590	31	8965	13	93.4	6.2	6.5	0.29	+4.8
High HDI	Mexico	12030	862	47	11 081	40	92.1	7.2	7.6	0.34	+4.7
	Lebanon	3911	280	14	3609	8	92.3	7.2	7.5	0.32	+4.4
	Peru	14 157	850	114	13 155	38	92.9	6.1	6.8	0.74	+12.2
	Brazil	6934	685	22	6221	6	89.7	9.9	10.2	0.28	+2.8
	Ecuador	9550	589	46	8886	29	93.0	6.2	6.6	0.43	+7.0
	Sri Lanka	17 877	1246	67	16 528	36	92.5	7.0	7.3	0.33	+4.8
Medium HDI	Jordan	1124	108	6	1009	1	89.8	9.7	10.1	0.47	+4.9
	China	12 803	708	21	12 064	10	94.2	5.5	5.7	0.15	+2.7
	Thailand	8858	870	35	7936	17	89.6	9.9	10.2	0.34	+3.4
	Mongolia	7249	328	24	6879	18	94.9	4.6	4.9	0.30	+6.7
	OPT	950	82	4	863	1	90.8	8.7	9.1	0.38	+4.3
	Paraguay	3571	271	6	3291	3	92.2	7.6	7.8	0.15	+2.0
	Philippines	10 494	767	54	9621	52	91.7	7.4	7.8	0.44	+6.0
	Vietnam	13 076	419	13	12639	5	96.7	3.2	3.3	0.10	+3.0
	Nicaragua	6346	525	24	5785	12	91.2	8.3	8.7	0.33	+4.0
	India	27 776	2903	529	23 870	474	85.9	10.8	12.4	1.51	+14.0
	Cambodia	4616	227	43	4316	30	93.5	5.0	5.8	0.85	+17.1
Low HDI	Kenya	19 845	1284	306	17 942	313	90.4	6.7	8.0	1.33	+20.0
	Pakistan	11 927	1034	216	10 528	149	88.3	8.9	10.5	1.54	+17.2
	Angola	3768	209	29	3432	98	91.1	5.7	6.3	0.58	+10.0
	Nigeria	8750	565	129	7855	201	89.8	6.7	7.9	1.22	+18.2
	Nepal	11 064	547	110	10 289	118	93.0	5.0	5.9	0.89	+17.6
	Uganda	4072	107	42	3830	93	94.1	2.7	3.7	0.94	+34.6
	Afghanistan	9340	142	98	8992	108	96.3	1.6	2.6	1.01	+65.3
	DRC	5402	361	45	4859	137	89.9	6.9	7.5	0.60	+8.7
	Niger	5751	108	54	5406	183	94.0	2.0	2.8	0.86	+43.8
All countries		258 215	17 004	2144	236 862	2205	91.7	6.7	7.4	0.72	+10.7

DRC, Democratic Republic of Congo; HDI, Human Development Index; OPT, occupied Palestine territory.

a Calculated as B – A: A – birth rate (per 100 deliveries) excluding live births, B – birth rate (per 100 deliveries) including stillbirths.

b Calculated 5 as (B – A)/A*100 (%): A – birth rate excluding live births, B – birth rate including stillbirth.

**Table 2:** Gestational age‐specific birth rates calculated including versus excluding stillbirths among countries of high‐, medium‐ and low‐Human Development Index participating in the WHO MultiCountry Survey

	HDI	Excluding stillbirths (per 1000 deliveries)		Including stillbirths (per 1000 deliveries)		Increase in rates by including stillbirths (%)^a^		Increase in rates by including stillbirths (%)^b^	
		Median	IQR	Median	IQR	Median	IQR	Median	IQR
22–27 weeks	Very high & high	1.7	0.5–2.6	3.3	1.7– 3.5	0.09	0.06–0.11	49.3	32.8–127.5
	Medium	2.0	0.8–3.6	3.7	1.7–4.2	0.09	0.03–0.12	49.1	24.3–96.8
	Low	1.1	0.9–1.6	3.3	2.5–3.6	0.21	0.17–0.31	188.7	142.3–222.3
28–31 weeks	Very high & high	5.4	4.9–6.9	6.6	6.1–7.4	0.10	0.07–0.13	20.6	8.3–22.7
	Medium	7.8	6.5–11.7	9.1	6.7–13.2	0.11	0.05–0.17	19.3	8.5–24.8
	Low	6.2	2.8–7.4	9.4	5.6–14.2	0.37	0.29–0.51	66.2	54.9–92.7
32–33 weeks	Very high & high	9.1	6.7–9.6	10.2	7.2–10.2	0.06	0.05–0.07	7.3	5.8–9.0
	Medium	11.8	7.3–13.8	12.9	7.7–15.8	0.06	0.04–0.20	5.4	3.7–14.6
	Low	6.8	3.3–7.8	7.4	5.5–9.9	0.19	0.09–0.22	28.8	24.8–45.9
34–36 weeks	Very high & high	47.9	41.0–54.8	48.4	41.9–55.5	0.07	0.05–0.09	1.7	1.1–1.8
	Medium	53.6	31.9–63.6	54.0	32.4–64.9	0.07	0.04–0.11	1.2	0.8–2.2
	Low	46.6	21.1–51.6	50.1	22.6–53.6	0.20	0.15–0.24	7.1	3.8–7.5
22–36 weeks	Very high & high	62.2	60.7–72.0	68.1	64.7–75.2	0.32	0.28–0.34	4.8	4.4–5.5
	Medium	76.1	50.0–96.7	78.2	56.9–101.4	0.34	0.15–0.47	4.3	3.0–6.7
	Low	57.4	27.2–67.1	63.2	36.6–79.3	0.94	0.86–1.22	18.2	17.2–34.6
		**(per 100 deliveries of at least 28 weeks)**		**(per 100 deliveries of at least 28 weeks)**		**(per 100 deliveries of at least 28 weeks)**		**(per 100 deliveries of at least 28 weeks)**	
28–36 weeks	Very high & high	61.7	58.0–69.8	64.9	62.0–72.1	0.21	0.19–0.30	3.6	2.1–5.3
	Medium	72.7	48.7–93.4	74.8	56.0–97.4	0.25	0.14–0.39	3.4	2.6–5.1
	Low	56.4	26.4–66.0	61.2	34.2–76.1	0.71	0.52–1.00	14.0	8.5–47.5

HDI, Human Development Index; IQR, interquartile range.

a Calculated as B – A: A – birth rate (per 100 deliveries) excluding live births, B – birth rate (per 100 deliveries) including stillbirths.

b Calculated 5 as (B – A)/A*100 (%): A – birth rate excluding live births, B – birth rate including stillbirth.

Preterm birth rates were lowest in low‐HDI countries, compared with medium‐ and very high/high‐HDI countries, regardless of inclusion/exclusion of stillbirths. However, the proportion of term live births among all deliveries was lower among low‐HDI countries (91.1%, 90.0–94.0%) than among very high/high‐HDI countries (93.2%, 92.7–93.6%).

## Discussion

### Main findings

The preterm birth rate has long been an important indicator of perinatal population health. For many countries, achievement of the Millennium Developmental Goal 4 was strongly influenced by progress in reducing neonatal deaths, of which preterm birth is the leading cause.^2^ Many initiatives^7^, ^28^ have aimed to develop and evaluate interventions to reduce preterm birth rates. However, our study suggests that without simultaneous evaluation of stillbirth rates, the true picture of preterm birth may be obscured. In this cross‐sectional study of a sample of deliveries in 29 countries, we found that inclusion of stillbirths substantially increases the preterm birth rate in all countries. The degree of change was particularly large in low‐HDI countries, with preterm births increasing by over 8% for all nine such countries when stillbirths were included.

### Strengths and limitations

The main strengths of our study include its large sample size, multicountry context, and uniformity in study methods and definitions of study variables collected across countries. However, our study also has several limitations. First, recorded gestational age was based on the best obstetric estimate, the basis of which was not recorded. The validity of gestational age estimation was questionable in some health facilities, leading to the exclusion of 22 facilities that reported over 70% of all births to have occurred during one gestational week. We attempted to minimise bias due to errors in gestational age estimation: first by restricting to health facilities with plausible gestational age distributions, and secondly by restricting our analysis to births with weights compatible with their gestational age. We also conducted a sensitivity analysis based on birthweight, rather than gestational age, to confirm our findings. Another limitation is that our study sample was representative only of those facilities with at least 1000 births per year and capable of providing caesarean delivery; in addition, our database includes home births. These features may explain why preterm estimates in many low‐income countries were low even when stillbirths were included. As the rates of stillbirths and preterm births are higher among home deliveries in low‐income countries, their inclusion would likely further increase the impact of stillbirths on preterm birth rates in those countries.

### Interpretation

The first WHO estimates of stillbirth were developed in 2006, highlighting the lack of available data, when two studies concurrently estimated 3 million stillbirths per year globally,^29^, ^30^ with 99% of them in low‐ and middle‐income countries. The absence of national registration systems for stillbirths, which had hidden this large global burden for so long, has now been addressed to some extent; the number of countries without national‐level stillbirth data was reduced to 38 in 2015.^31^ However, a recent review reported the number of stillbirths had reduced only slightly to an estimated 2.6 million globally in 2015, with death registration provided for fewer than 5% of these deaths.^9^, ^32^, ^33^ Further improvement in stillbirth registration and reporting will enable both accurate temporal monitoring and international comparisons of perinatal health.

Previous studies have estimated national and regional rates of both preterm birth and stillbirth.^9^, ^12^ However, as stillbirth reporting is usually aggregated and not reported according to gestational age, previous international comparisons of preterm birth and stillbirth rates were not able to evaluate the extent to which stillbirths affect preterm birth rates. One study involving 193 countries reported that low birthweight rates of up to about 10% are positively correlated with stillbirth rates, especially in low‐ and middle‐income countries,^34^ whereas another study of 28 high‐income countries found preterm birth rates to be inversely associated with stillbirth rates.^35^ These findings are in line with our finding that misclassification of vital status can affect preterm birth rates, and that given the large variation in stillbirth rates between countries, the current preterm rate should not be interpreted without also considering the stillbirth rate.

Very few studies have investigated how stillbirths affect the preterm birth rate. To the best of our knowledge, our recent study on 30 high‐income countries is the only previous study investigating this issue.^36^ We found that in high income‐countries, although very preterm birth rates were higher when stillbirths were included, their inclusion did not substantially affect international ranking of preterm birth rates. The study we report herein is the only one from middle‐ and low‐income countries and shows that the impact of stillbirth is much larger, and thus stillbirth registration is therefore of greater importance, in low‐income countries. The differences in our two studies are most likely due to less access to intensive fetal monitoring or provider‐initiated delivery when at risk of fetal death, as well as more misclassification of neonatal deaths as stillbirths, in low‐income countries. We believe the true impact may be even larger in some countries, as our study did not capture home deliveries, which are far more frequent in such countries.^37^

In addition to underscoring the importance of stillbirth registration for monitoring perinatal health, our study also provides information that could help estimate the preterm delivery rate among all births in countries where stillbirth rates are not provided. The increase in preterm birth rates when including stillbirths varied to some extent among countries, although patterns were observed in relation to country HDI. The range for very high‐/high‐/medium‐HDI countries was +2.0 to +12.2% (with the exception of India: +14.0% and Cambodia: +17.1%, which have the lowest HDI in the middle‐HDI category). For low‐HDI countries, this range was +8.7 to +65.3%. Low‐HDI countries in South Asia and sub‐Saharan Africa are reported to have the largest burdens of both preterm births^12^ and stillbirths,^9^ and their preterm births are likely to increase by at least 8% when stillbirths are included. This is important for international comparisons of preterm birth data, as our findings can also be used to estimate the impact of stillbirths on preterm birth rates in settings where details on stillbirth data are not available.

## Conclusion

We have shown not only that the stillbirth rate is an important perinatal health indicator in and of itself, but that stillbirths substantially affect other important indicators such as preterm delivery. Preterm birth rates based on live births alone do not accurately reflect international disparities in perinatal health. Improved registration and reporting of stillbirths should increase support for the infrastructure and training necessary to monitor their occurrence, and should also improve the validity of international comparisons of perinatal health.

### Disclosure of interests

None declared. Completed disclosure of interests form available to view online as supporting Information.

### Contribution to authorship

NM and TG contributed equally. JV, MSK and NM initiated the concept. NM, TG, MSK, JV and JZ designed the study. TG and NM performed the analysis. NM and TG drafted the initial manuscript, and MSK provided oversight of the process. JGC, JPS, CPC, MRT, EO, RM, SMD, SM, VB, BY and ST reviewed and approved the submitted manuscript.

### Details of ethics approval

The UNDP/UNFPA/UNICEF/WHO/World Bank Special Programme of Research, Development and Research Training in Human Reproduction (HRP) Specialist Panel on Epidemiological Research reviewed and approved the study protocol for technical content. This study was approved by the World Health Organization Ethical Review Committee (A65661, date 27 October 2009) and the relevant ethical clearance mechanisms in all countries.

### Funding

UNDP/UNFPA/UNICEF/WHO/World Bank Special Programme of Research, Development and Research Training in Human Reproduction; WHO; USAID; Ministry of Health, Labor and Welfare of Japan. This paper represents the views of the named authors only, not the views of their institutions or organisations.

## Supporting information


**Table S1**. Number of health facilities and deliveries excluded, and study population by participated countries.

**Table S2**. Proportion of live births by gestational age, among 29 countries participating in the WHO Multicountry Survey.

**Table S3**. Percentage of live births among deliveries, stratified by gestational age. Comparison between countries of high‐, medium‐ and low‐ Human Developmental Index participating in the WHO Multicountry Survey.

**Table S4**. Percentage of live births among deliveries, stratified by birthweight. Comparison between countries of high‐, medium‐ and low‐Human Developmental Index participating in the WHO Multicountry Survey.

**Table S5**. Percentage of live births among deliveries including multiple births, stratified by gestational age. Comparison between countries of high‐, medium‐ and low‐Human Developmental Index participating in the WHO Multicountry Survey.

**Table S6**. Birthweight specific rates calculated including and excluding stillbirths, among countries of high‐, medium‐ and low‐Human Development index.

**Table S7**. Gestational age‐specific birth rates calculated including and excluding stillbirths, among countries of high‐, medium‐ and low‐Human Development index. Analysis including multiple births.
