# Supplementary material for: Impact of stillbirths on international comparisons of preterm birth rates: a secondary analysis of the WHO multi‐country survey of Maternal and Newborn Health
Source: BJOG. 2017 Feb 20;124(9):1346–54. doi: 10.1111/1471-0528.14548 (PMC5573985; doi:10.1111/1471-0528.14548)
Supplement: Supplementary file 1 — Table S1. Number of health facilities and deliveries excluded, and study population by participated countries. [file BJO-124-1346-s001.pdf]

Table S1. Number of health facilities and deliveries excluded, and study population by participated countries.

HDI groups	Country	Facility	Delivery	Exclusion of health facilities: <100 deliveries — Facility	Delivery	>5% missing gestational age — Facility	Delivery	>70% have same gestation — Facility	Delivery	Preterm delivery >30% — Facility	Delivery	Preterm delivery <1% — Facility	Delivery	Exclusion of individual deliveries	Total excluded: Facility n	,%	Delivery	n,%	Study population: Facility	Delivery
Very high	Japan	10	3,537											41	0	0	41	1.2	10	3,496
	Qatar	1	3,950											71	0	0.0	71	1.8	1	3,879
	Argentina	14	9,807											208	0	0	208	2.1	14	9,599
	Mexico	14	13,309							1	1108			171	1	7.1	1,279	9.6	13	12,030
	Lebanon	9	4,044											133	0	0	133	3.3	9	3,911
High	Peru	16	15,285			1	544					2	325	259	3	18.8	1,128	7.4	13	14,157
	Brazil	7	7,058											124	0	0	124	1.8	7	6,934
	Ecuador	18	10,245	6	386									309	6	33.3	695	6.8	12	9,550
	Sri Lanka	14	18,129											252	0	0.0	252	1.4	14	17,877
	Jordan	1	1,167											43	0	0	43	3.7	1	1,124
	China	21	13,277	1	97							1	187	190	2	9.5	474	3.6	19	12,803
	Thailand	12	8,973											115	0	0.0	115	1.3	12	8,858
	Mongolia	5	7,365											116	0	0	116	1.6	5	7,249
	OPT	1	980											30	0	0.0	30	3.1	1	950
Medium	Paraguay	6	3,610											39	0	0.0	39	1.1	6	3,571
	Philippines	16	10,783	2	2									287	2	12.5	289	2.7	14	10,494
	Viet Nam	15	15,437					1	238			4	1954	169	5	33.3	2,361	15.3	10	13,076
	Nicaragua	8	6,571	2	46									179	2	25	225	3.4	6	6,346
	India	21	31,318					2	2780					762	2	9.5	3,542	11.3	19	27,776
	Cambodia	5	4,725											109	0	0	109	2.3	5	4,616
	Kenya	20	20,354											509	0	0	509	2.5	20	19,845
	Pakistan	16	13,175					1	151	1	369	1	497	231	3	18.8	1,248	9.5	13	11,927
	Angola	20	10,450	4	168	1	218	7	6069			1	128	99	12	60	6,682	63.9	7	3,768
	Nigeria	21	12,841			3	2056	2	1503					532	5	23.8	4,091	31.9	16	8,750
Low	Nepal	8	11,290											226	0	0	226	2.0	8	11,064
	Uganda	20	10,923	8	412	3	6115					1	115	209	12	60.0	6,851	62.7	8	4,072
	Afghanistan	8	26,148					6	16593					215	6	75	16,808	64.3	2	9,340
	DRC	21	8,756			5	2951			1	205			198	6	28.6	3,354	38.3	15	5,402
	Niger	11	11,116					3	2964			2	2176	225	5	45.5	5,365	48.3	6	5,751
All		359	314,623	23	11	13	11884	22	30298	3	1682	12	5382	7,151	73	20.3	56,408	17.9	286	258,215

Country ordered from high to low Human Development index of 2012
