# Supplementary material for: Impact of stillbirths on international comparisons of preterm birth rates: a secondary analysis of the WHO multi‐country survey of Maternal and Newborn Health
Source: BJOG. 2017 Feb 20;124(9):1346–54. doi: 10.1111/1471-0528.14548 (PMC5573985; doi:10.1111/1471-0528.14548)
Supplement: Supplementary file 2 — Table S2. Proportion of live births by gestational age, among 29 countries participating in the WHO Multicountry Survey. [file BJO-124-1346-s002.pdf]

Table S2. Proportion of live births by gestational age, among 29 countries participating in the WHO multi-country survey

		<28 weeks			28-31 weeks			32-33 weeks			34-36 weeks		
		**LB**	**SB**	**%**	**LB**	**SB**	**%**	**LB**	**SB**	**%**	**LB**	**SB**	**%**
Very High	Japan	10	2	83.3	17	0	100.0	18	1	94.7	142	3	97.9
	Qatar	1	0	100.0	4	4	50.0	4	2	66.7	141	3	97.9
	Argentina	16	8	66.7	52	11	82.5	64	5	92.8	458	7	98.5
High	Mexico	20	26	43.5	83	6	93.3	125	5	96.2	634	10	98.4
	Lebanon	9	4	69.2	21	3	87.5	37	3	92.5	213	4	98.2
	Peru	40	52	43.5	108	26	80.6	128	17	88.3	574	19	96.8
	Brazil	18	6	75.0	80	7	92.0	95	6	94.1	492	3	99.4
	Ecuador	5	11	31.3	55	13	80.9	91	6	93.8	438	16	96.5
	Sri Lanka	9	6	60.0	87	25	77.7	135	13	91.2	1,015	23	97.8
Medium	Jordan	4	1	80.0	10	2	83.3	23	1	95.8	71	2	97.3
	China	2	1	66.7	55	12	82.1	93	5	94.9	558	3	99.5
	Thailand	18	8	69.2	69	12	85.2	99	3	97.1	684	12	98.3
	Mongolia	6	6	50.0	47	10	82.5	45	3	93.8	230	5	97.9
	OPT	4	1	80.0	7	0	100.0	13	2	86.7	58	1	98.3
	Paraguay	13	1	92.9	23	1	95.8	44	2	95.7	191	2	99.0
	Philippines	26	13	66.7	108	13	89.3	123	12	91.1	510	16	97.0
	Viet Nam	6	4	60.0	24	6	80.0	57	2	96.6	332	1	99.7
	Nicaragua	16	8	66.7	77	7	91.7	67	4	94.4	365	5	98.6
	India	43	73	37.1	339	200	62.9	461	87	84.1	2,060	169	92.4
	Cambodia	6	6	50.0	53	19	73.6	54	11	83.1	114	7	94.2
Low	Kenya	32	34	48.5	179	107	62.6	177	67	72.5	896	98	90.1
	Pakistan	30	45	40.0	85	84	50.3	132	38	77.6	787	49	94.1
	Angola	4	4	50.0	11	9	55.0	6	2	75.0	188	14	93.1
	Nigeria	10	20	33.3	79	51	60.8	66	21	75.9	410	37	91.7
	Nepal	10	23	30.3	67	39	63.2	74	11	87.1	396	37	91.5
	Uganda	3	7	30.0	11	20	35.5	10	6	62.5	83	9	90.2
	Afghanistan	5	29	14.7	17	35	32.7	30	21	58.8	90	13	87.4
	DRC	5	8	38.5	34	17	66.7	37	3	92.5	285	17	94.4
	Niger	9	19	32.1	15	11	57.7	23	12	65.7	61	12	83.6
All regions	Total	380	426	47.1	1,817	750	70.8	2,331	371	86.3	12,476	597	95.4

LB: Number of live births, SB: Number of stillbirths, %: percentage of live births among live + stillbirths
