# Supplementary material for: Impact of stillbirths on international comparisons of preterm birth rates: a secondary analysis of the WHO multi‐country survey of Maternal and Newborn Health
Source: BJOG. 2017 Feb 20;124(9):1346–54. doi: 10.1111/1471-0528.14548 (PMC5573985; doi:10.1111/1471-0528.14548)
Supplement: Supplementary file 3 — Table S3. Percentage of live births among deliveries, stratified by gestational age. Comparison between countries of high‐, medium‐ and low‐ Human Developmental Index participating in the WHO Multicountry Survey. [file BJO-124-1346-s003.pdf]

Table S3. Percentage of live births among deliveries, stratified by gestational age. Comparison between countries of high, medium and low Human Developmental Index participating in the WHO multi-country survey

GA	HDI	Number of births		Percentage of live births among deliveries		Between 3 groups*	Between Very High/High and Medium HDI*	Between Medium and Low HDI*
		live births	stillbirths	Median	IQR			
22-23 weeks	Very high/High	19	48	36.7	20.2-45.0	p=0.201	p=0.824	p=0.247
	Medium	16	31	32.7	0.0-50.0			
	Low	9	38	0.0	0.0-25.0			
24-27 weeks	Very high/High	109	67	73.7	62.1-83.3	p=0.001	p=0.676	p<0.001
	Medium	128	91	73.3	60.0-80.0			
	Low	99	151	38.5	33.3-44.8			
28-31 weeks	Very high/High	507	95	82.5	80.6-91.9	p=0.001	p=0.939	p<0.001
	Medium	812	282	83.3	80.0-91.7			
	Low	498	373	57.7	50.3-62.6			
32-33 weeks	Very high/High	697	58	92.8	91.2-94.1	p=0.002	p=0.470	p=0.001
	Medium	1,079	132	94.4	86.7-95.8			
	Low	555	181	75.0	65.7-77.6			
34-36 weeks	Very high/High	4,107	88	97.9	97.8-98.4	p<0.001	p=0.790	p<0.001
	Medium	5,173	223	98.3	97.0-99.0			
	Low	3,196	286	91.5	90.1-93.1			

HDI, Human Development Index; WHO, World Health Organization; IQR, Interquartile range
* Kruskal-Wallis rank test used
