# Supplementary material for: Impact of stillbirths on international comparisons of preterm birth rates: a secondary analysis of the WHO multi‐country survey of Maternal and Newborn Health
Source: BJOG. 2017 Feb 20;124(9):1346–54. doi: 10.1111/1471-0528.14548 (PMC5573985; doi:10.1111/1471-0528.14548)
Supplement: Supplementary file 4 — Table S4. Percentage of live births among deliveries, stratified by birthweight. Comparison between countries of high‐, medium‐ and low‐Human Developmental Index participating in the WHO Multicountry Survey. [file BJO-124-1346-s004.pdf]

Table S4. Percentage of live births among deliveries, stratified by birthweight. Comparison between countries of high, medium and low Human Developmental Index participating in the WHO multi-country survey

Birth weight	HDI	Percentage of live births among deliveries		Between 3 groups*	Between Very high/High and Medium HDI countries*	Between Medium vs Low HDI countries*
		Median	IQR			
<1000	Very High/High	55.4	50.0-69.2			
	Medium	58.8	46.2-70.5	p<0.001	p=0.969	p=0.001
	Low	32.4	18.2-36.4			
1000-1500	Very High/High	89.3	80.8-91.1			
	Medium	83.9	75.9-90.0	p<0.001	p=0.518	p<0.001
	Low	58.6	55.3-62.5			
1500-2000	Very High/High	93.5	92.5-95.1			
	Medium	93.9	90.1-96.5	p<0.001	p=0.732	p<0.001
	Low	79.4	72.5-83.2			
2000-2500	Very High/High	98.5	97.7-99.5			
	Medium	98.7	96.2-100.0	p<0.001	p=0.849	p=0.001
	Low	93.6	90.6-95.1			

HDI, Human Development Index; WHO, World Health Organization; IQR, Interquartile range

* Kruskal-Wallis rank test used
