# Supplementary material for: Impact of stillbirths on international comparisons of preterm birth rates: a secondary analysis of the WHO multi‐country survey of Maternal and Newborn Health
Source: BJOG. 2017 Feb 20;124(9):1346–54. doi: 10.1111/1471-0528.14548 (PMC5573985; doi:10.1111/1471-0528.14548)
Supplement: Supplementary file 5 — Table S5. Percentage of live births among deliveries including multiple births, stratified by gestational age. Comparison between countries of high‐, medium‐ and low‐Human Developmental Index participating in the WHO Multicountry Survey. [file BJO-124-1346-s005.pdf]

Table S5. Percentage of live births among deliveries including multiple births, stratified by gestational age. Comparison between countries of high, medium and low Human Developmental Index participating in the WHO multi-country survey.

Gestational age	HDI	Number of births		Percentage of live births among deliveries		Between 3 groups*	Between Very High/High and Medium HDI*	Between Medium and Low HDI*
		LB	All	Median	IQR			
22-23 weeks	Very high/High	20	68	36.7	23.4-50.0	p=0.182	p=0.637	p=0.298
	Medium	16	49	30.5	0.0-47.2			
	Low	12	57	0.0	0.0-25.0			
24-27 weeks	Very high/High	116	185	75.0	64.7-83.3	p=0.002	p=0.425	p=0.001
	Medium	131	223	72.7	60.0-80.0			
	Low	135	294	46.7	35.0-50.0			
28-31 weeks	Very high/High	560	659	85.7	81.8-92.9	p<0.001	p=0.879	p<0.001
	Medium	884	1,169	86.7	81.3-92.6			
	Low	710	1,107	62.1	54.8-65.0			
32-33 weeks	Very high/High	765	825	94.2	92.2-95.6	p=0.002	p=0.645	p=0.001
	Medium	1,170	1,304	95.2	88.2-96.1			
	Low	841	1,040	79.0	70.7-80.0			
34-36 weeks	Very high/High	4,490	4,582	98.4	97.8-98.7	p<0.001	p=0.849	p<0.001
	Medium	5,535	5,764	98.1	97.0-99.1			
	Low	4,282	4,600	92.1	90.4-93.2			

HDI, Human Development Index; WHO, World Health Organization; IQR, Interquartile range
