# Supplementary material for: Impact of stillbirths on international comparisons of preterm birth rates: a secondary analysis of the WHO multi‐country survey of Maternal and Newborn Health
Source: BJOG. 2017 Feb 20;124(9):1346–54. doi: 10.1111/1471-0528.14548 (PMC5573985; doi:10.1111/1471-0528.14548)
Supplement: Supplementary file 6 — Table S6. Birthweight specific rates calculated including and excluding stillbirths, among countries of high‐, medium‐ and low‐Human Development index. [file BJO-124-1346-s006.pdf]

Table S6. Birth weight specific rates calculated including and excluding stillbirths, among countries of high, medium and low Human Development index.

	HDI	Excluding stillbirths (per 100 deliveries)		Including stillbirths (per 100 deliveries)		Increase in rates by including stillbirths (%)#	
		Median	IQR	Median	IQR	Median	IQR
<1000 grams	Very High & High	0.3	0.2-0.3	0.4	0.3- 0.5	79.3	43.8-99.1
	Medium	0.2	0.1-0.3	0.4	0.2-0.5	69.0	40.5-115.4
	Low	0.1	0.0-0.1	0.3	0.2-0.4	196.7	169.3-431.5
1000-1499 grams	Very High & High	0.6	0.5-0.6	0.7	0.6-0.8	11.5	9.3-23.1
	Medium	0.8	0.3-1.0	0.8	0.4-1.1	18.0	10.5-31.1
	Low	0.5	0.4-0.7	0.8	0.7-1.1	64.8	55.0-73.3
1500-1999 grams	Very High & High	1.3	1.2-1.6	1.4	1.3-1.7	6.2	4.4-7.6
	Medium	1.5	0.8-2.0	1.6	0.8-2.2	5.7	3.1-9.4
	Low	1.5	0.7-1.7	1.9	1.0-1.9	21.7	15.6-33.6
2000-2499 grams	Very High & High	6.0	4.0-7.7	6.0	4.0-7.7	0.8	0.1-1.6
	Medium	6.0	3.8-7.2	6.2	3.8-7.2	0.4	0.0-1.1
	Low	6.3	4.5-7.5	6.5	4.8-7.7	3.4	1.8-5.8
<2500 grams	Very High & High	9.1	5.7-9.5	9.4	6.4-9.7	4.2	2.8-5.6
	Medium	8.7	4.6-10.6	9.4	4.7-10.9	3.6	2.9-5.6
	Low	8.7	6.9-10.4	10.0	7.6-12.1	13.7	8.8-16.1
		(per 100 deliveries of at least 1000 grams)		(per 100 deliveries of at least 1000 grams)			
1000-2500 grams	Very High & High	8.8	5.3-9.3	8.9	5.7-9.4	2.9	1.5-3.6
	Medium	8.5	4.5-9.9	9.1	4.6-10.1	2.3	1.7-4.5
	Low	8.6	6.8-10.3	9.5	7.5-11.6	11.8	8.3-16.0

HDI, Human Development Index; WHO, World Health Organization; IQR, Interquartile range

calculated as (B-A)/A*100 (%) with A:birth rate excluding live births, B:birth rate including stillbirths
