# Supplementary material for: Impact of stillbirths on international comparisons of preterm birth rates: a secondary analysis of the WHO multi‐country survey of Maternal and Newborn Health
Source: BJOG. 2017 Feb 20;124(9):1346–54. doi: 10.1111/1471-0528.14548 (PMC5573985; doi:10.1111/1471-0528.14548)
Supplement: Supplementary file 7 — Table S7. Gestational age‐specific birth rates calculated including and excluding stillbirths, among countries of high‐, medium‐ and low‐Human Development index. Analysis including multiple births. [file BJO-124-1346-s007.pdf]

Table S7. Gestational-age-specific birth rates calculated including and excluding stillbirths, among countries of high, medium and low Human Development index. Analysis including multiple births.

	HDI	Excluding stillbirths (per 100 deliveries)		Including stillbirths (per 100 deliveries)		Increase in rates by including stillbirths (%)[#]	
		**Median**	**IQR**	**Median**	**IQR**	**Median**	**IQR**
22-27 weeks	Very High & High	0.2	0.1- 0.3	0.3	0.2-0.4	45.8	31.0-113.8
	Medium	0.2	0.1-0.3	0.4	0.2-0.4	49.6	24.4-96.8
	Low	0.1	0.1-0.2	0.4	0.3-0.4	140.3	93.3-198.2
28-31 weeks	Very High & High	0.7	0.6-0.9	0.8	0.7-1.1	16.1	7.1-20.9
	Medium	1.0	0.6-1.3	1.1	0.6-1.3	14.6	73.-22.9
	Low	0.7	0.3-0.9	1.0	0.6-1.5	54.4	48.6-76.8
32-33 weeks	Very High & High	1.0	0.8-1.2	1.1	0.9-1.2	5.5	4.3-7.8
	Medium	1.3	0.8-1.5	1.4	0.8-1.7	4.5	3.4-12.5
	Low	0.8	0.4-1.1	0.9	0.6-1.3	21.8	20.8-35.5
34-36 weeks	Very High & High	5.9	4.8-6.3	6.0	4.9-6.4	1.1	0.7-1.9
	Medium	5.9	3.9-8.2	5.9	4.0-8.3	1.3	0.6-2.1
	Low	5.2	2.3-5.4	5.4	2.5-5.7	6.0	3.7-6.9
22-36 weeks	Very High & High	7.6	6.4-8.2	7.9	6.9-8.6	4.1	3.4-4.5
	Medium	8.2	5.7-10.6	8.3	6.5-11.0	3.5	2.6-5.6
	Low	5.8	3.0-7.6	6.6	4.0-8.8	16.0	15.6-30.1
		(per 100 deliveries above 28 weeks)		(per 100 deliveries above 28 weeks)			
28-36 weeks*	Very High & High	7.4	6.4-8.0	7.6	6.7-8.1	2.5	1.9-4.1
	Medium	7.9	5.6-10.5	7.9	6.3-10.7	2.5	1.9-4.2
	Low	5.7	2.9-7.4	6.3	3.7-8.4	13.2	11.8-23.4

calculated as (B-A)/A*100 (%) with A:birth rate excluding live births, B:birth rate including stillbirth
